# Orthologous MicroRNA Genes Are Located in Cancer-Associated Genomic Regions in Human and Mouse

**DOI:** 10.1371/journal.pone.0001133

**Published:** 2007-11-07

**Authors:** Igor V. Makunin, Michael Pheasant, Cas Simons, John S. Mattick

**Affiliations:** Australian Research Council (ARC) Special Research Centre for Functional and Applied Genomics, Institute for Molecular Bioscience, University of Queensland, Brisbane, Queensland, Australia; Sanofi-Aventis, United States of America

## Abstract

**Background:**

MicroRNAs (miRNAs) are short non-coding RNAs that regulate differentiation and development in many organisms and play an important role in cancer.

**Methodology/Principal Findings:**

Using a public database of mapped retroviral insertion sites from various mouse models of cancer we demonstrate that MLV-derived retroviral inserts are enriched in close proximity to mouse miRNA loci. Clustered inserts from cancer-associated regions (Common Integration Sites, CIS) have a higher association with miRNAs than non-clustered inserts. Ten CIS-associated miRNA loci containing 22 miRNAs are located within 10 kb of known CIS insertions. Only one CIS-associated miRNA locus overlaps a RefSeq protein-coding gene and six loci are located more than 10 kb from any RefSeq gene. CIS-associated miRNAs on average are more conserved in vertebrates than miRNAs associated with non-CIS inserts and their human homologs are also located in regions perturbed in cancer. In addition we show that miRNA genes are enriched around promoter and/or terminator regions of RefSeq genes in both mouse and human.

**Conclusions/Significance:**

We provide a list of ten miRNA loci potentially involved in the development of blood cancer or brain tumors. There is independent experimental support from other studies for the involvement of miRNAs from at least three CIS-associated miRNA loci in cancer development.

## Introduction

MicroRNAs (miRNAs) are short RNA molecules, ∼22 nucleotides long, capable of performing regulatory functions. In particular, miRNAs can suppress translation by non-perfect pairing to 3′ UTRs and/or cause degradation of mRNAs in the case of a perfect match between the miRNA and target mRNA [1]. It seems that miRNAs do not have any catalytic activity but rather act as sequence-specific guides for associated protein complexes which are responsible for translation suppression or degradation of mRNA [2]. The number of known miRNAs is growing rapidly, and hundreds of verified miRNAs are annotated in human, mouse, and other organisms (miRBase, http://microrna.sanger.ac.uk).

A range of observations point to a link between miRNAs and cancer, which is not surprising given their central role in many cellular and developmental processes (for reviews see [3]–[5]). A large number of human and mouse miRNAs have also been shown to be located in regions associated with cancer [6], [7]. The expression of various miRNAs is altered in cancer and miRNA profiling can be used for precise cancer classification [8]. Ectopic expression of the *mir-17-19b* cluster accelerates tumor formation in mice and has been accordingly classified as a potential oncogene [9].

Retroviruses, such as the murine leukemia virus (MLV), can cause tumor formation in mammals. Proviral insertions may activate proto-oncogenes or lead to inactivation of tumor suppressor genes in the vicinity of the insertion sites. Retroviral integration sites can be determined in animals with cancer using inverse PCR or similar techniques, and mapped to the genome sequence. Regions harboring multiple insertion sites in close proximity to each other are the most obvious candidates for cause of cancer development and are often named Common Integration Sites (CISs). In general, candidates for tumor-suppressor genes or proto-oncogenes are selected from protein-coding genes based on proximity to CISs.

Numerous genome-wide screens have been undertaken in mouse to identify genes involved in carcinogenesis, especially hematopoietic tumors [10]–[18]. Data obtained from various screens on different cancer models (including studies with genetically modified mice) has been compiled into the Retroviral Tagged Cancer Gene Database (RTCGD, http://rtcgd.ncifcrf.gov) [19] which also provides annotations for the UCSC genome browser [20]. In the RTCGD, the CISs are defined as regions containing two inserts located within 20 kb, 3 inserts within 50 kb, and four or more inserts within 100 kb in the same cancer model (see FAQ section of RTCGD on http://rtcgd.ncifcrf.gov) (for details see [17]).

However, some CISs do not map near any known or annotated protein-coding sequence. It was shown that insertions of retroviruses in the vicinity of the *mir-17-92* miRNA polycistron cause tumor formation and increase miRNA expression, indicating that retroviral mutagenesis can be a potent tool for discovery of oncogenic miRNAs [21], [22]. Considering the emerging regulatory role of microRNAs in cell differentiation and cancer we analyzed the association between publicly available retroviral integration sites and known miRNA loci in the mouse genome. We found that miRNA loci are significantly enriched in the vicinity of CISs which suggests that some of these miRNA loci may also be considered as candidate proto-oncogenes or tumor-suppressor genes.

## Results

### Murine miRNA loci associate with retroviral common integration sites

We analyzed co-localization between mouse miRNAs and retroviral integration sites determined from mice that developed cancer. For this analysis we used the 363 miRNAs from the miRNA registry (http://microrna.sanger.ac.uk) that have been mapped to 381 locations within the well-assembled fraction of the mouse genome (four miRNAs mapped to more than one location). The locations correspond to the genomic positions of miRNA precursor sequences. We used the RTCGD database containing 2373 retroviral integration sites within Common Integration Sites (CIS inserts) and 3119 retroviral integration sites mapped outside of CISs (non-CIS inserts). We excluded from our analysis integration sites of Sleeping Beauty transposons because non-CIS insertions of Sleeping Beauty are non-randomly distributed among the chromosomes: chromosomes 1, 4, 6 and 15 harbor more than half of all Sleeping Beauty non-CIS integration sites.

**Figure 1:**
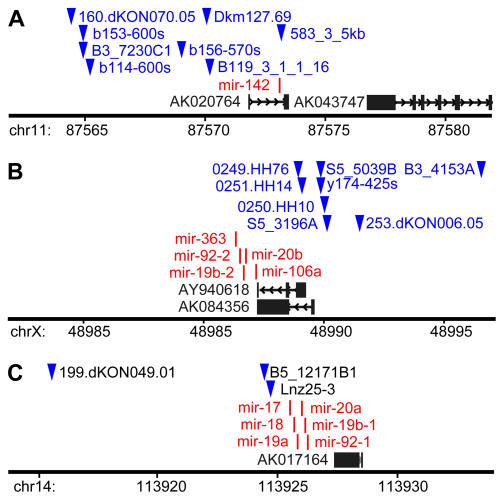
Examples of co-localization between miRNAs and CIS retroviral integration sites in the mouse genome. Each panel represents 20 kb of genomic DNA. Blue triangles indicate retroviral integration sites, red ticks represent miRNAs, black boxes and lines show the position of spliced transcripts. (A) chr11:87,562,001–87,582,000. (B) chrX:48,977,001–48,997,000. (C) chr14:113,914,001–113,934,000. Genome assembly mm8.

**Figure 2:**
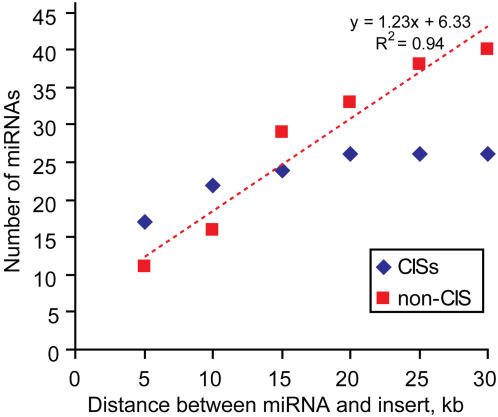
The number of miRNAs co-localized with retroviral integration sites. Blue diamonds represent the number of miRNAs located at the indicated distance or less from CIS inserts, and red diamonds correspond to miRNAs located at the indicated distance or less from non-CIS inserts. The trendline for miRNAs associated with non-CIS inserts is shown as red dotted line.

Using a local mirror of the UCSC genome browser we found that CIS inserts are located within 5 kb of 17 murine miRNAs and within 10 kb of 22 miRNAs. Examples of co-localization between miRNAs and CIS inserts are shown in Fig. 1 and a full list of CIS-associated miRNAs is given in Table 1. Further increasing the distance (past 10 kb) did not result in a significant increase of miRNA numbers associated with CIS inserts (Fig. 2) indicating that the association between miRNAs and CIS inserts is maximal at short distances.

**Table 1:** Retroviral CIS insertions within 10 kb of murine miRNAs.

MiRNA	Insertions^b^	Type^c^	RIS gene	Expression^c^	Human cancer
mir-9-1	* PDGFB.H8255 [12]	Brain tumor	Rhbg	Brain	
mir-196b a	* 403_0_8 [17]	myeloid	Hoxa7/Hoxa9	BM	
	* M15-5S8 [10]	B cell			
	* M35-5B3 [10]	B cell			
	* M17-5B5-1 [10]	B cell			
	* 45f23 [17]	myeloid			
	* 443_0_8 [17]	myeloid			
	* SL024-1 [18]	myeloid			
	* 143_1 [17]	T cell			
	* M35-5S2-1 [10]	B cell			
	* M8-5S2-1 [10]	B cell			
mir-135a-1	Dkm4.24 [15]	B cell	Wdr82	N/D	Lung, breast cancer
let-7g	Dkm83.2 [15]	B cell	Wdr82	Thymus	
mir-363	* 0249.HH76 [11]	T cell	Phf6	N/D	Advanced ovarian carcinoma
mir-92-2	* 0251.HH14 [11]	T cell		BM	
mir-19b-2	* S5_5039B [16]	B cell		HeLa, Colon	
mir-20b	* y174-425s [13]	Lymphoma			
mir-106a	* 0250.HH10 [11]	T cell		HeLa, BM	
	* S5_3196A [16]	B cell			
	* 253.dKON006.05 [11]	T cell			
	B3_4153A [16]	B cell			
mir-212	* T5_11746C1 [16]	B cell	Hic1/Dph1	Heart	Hepatocellular carcinoma, lung cancer
mir-132	* S5_7233D [16]	B cell		Frontal Cortex	
	* i142-4000p [13]	Lymphoma			
mir-22	* S3_049b [17]	B cell	2010305C02Rik	Skeletal Muscle	
	* S158_3_11_29 [17]	myeloid			
mir-142	160.dKON070.05 [11]	T cell	Supt4h2/Bzrap1	BM	Prolymphocytic leukemia, breast cancer
	b153-600s [13]	Lymphoma			
	B3_7230C1 [16]	B cell			
	b114-600s [13]	HS			
	* b156-570s [13]	Lymphoma			
	* Dkm127.69 [15]	B cell			
	* B119_3_1_1_16 [17]	myeloid			
	* 583_3_5kb [17]	N/D			
mir-23b	PDGFB.B42 [12]	Brain tumor	Fancc	Heart	Urothelial cancer
mir-27b				N/D	
mir-24-1				Placenta	
mir-17	199.dKON049.01 [11]	T cell	Gpc5	HeLa	Follicular lymphoma
mir-18	* B5_12171B1 [16]	B cell		HeLa, BM	
mir-19a	* Lnz25-3 [14]	T cell		HeLa, Colon	
mir-20a				HeLa, BM	
mir-19b-1				HeLa, Colon	
mir-92-1				BM	

a Not all RISs are shown.

b Reference in brackets, * less than 5 kb between insert and miRNA loci.

c N/D – not determined, BM – bone marrow, HS – histiocytic sarcoma.

We used a bootstrap simulation to estimate the statistical significance of the co-localization between retroviral integration sites and miRNAs (see Materials and Methods). Because some miRNAs are clustered in the genome and hence distributed non-uniformly, we grouped miRNAs into loci by adding 5, 10, 20 or 30 kb to each side of the miRNA location and combining the overlapping regions. This grouping is necessary to maintain clustered and tandemly repeated miRNAs as single units (loci) during the bootstrap procedure. Regions of the same sizes were randomly placed on the mouse genome and cases of overlap with retroviral integration sites were counted. The number of miRNA loci that are located 10 kb or less from CIS inserts is approximately 5.5 times higher than that observed for randomly placed loci, and the probability of obtaining such a number by chance is estimated as 1.7×10^−5^. The enrichment declines with the length of the miRNA loci, and the probability of obtaining a similar overlap between miRNA loci and CIS inserts by chance is higher for longer distances (Table 2). The bootstrap data indicate that the strongest association between miRNA loci and CIS inserts is at short distances, up to 10 kb. In agreement with this, the number of CIS retroviral inserts near to individual miRNAs is also highly enriched at short distances, and the enrichment declines with distance.

**Table 2:** Bootstrap analysis for co-localization between miRNAs and CIS inserts.

Distance, Kb	MiRNA loci, #	Observed^a^ # loci # inserts	Enrichment, fold^b^	Bootstrap (10^7^iterations)	
				P^c^	Max^d^
5	245	7	6.3	0.00014	10
		29	11.6	0.0034	118
10	237	10	5.5	0.000017	13
		44	9.3	0.0031	122
20	233	11	3.7	0.00021	15
		65	7.1	0.0044	166
30	228	11	2.8	0.0019	18
		77	6.1	0.0038	195

a Number of miRNA loci overlapping CIS inserts in the mouse genome.

b Ratio between observed and bootstrap average.

c Probability of obtaining at least the observed number of miRNA loci overlapping CIS inserts (upper line) or the probability of obtaining at least that number of CIS inserts overlapping miRNA loci (lower line) in bootstrap procedure.

d Maximum number of miRNA loci overlapping with CIS inserts (upper line) or maximum number of CIS inserts overlapping miRNA loci (lower line) obtained in the bootstrap procedure.

### Non-CIS inserts are enriched in the vicinity of miRNA loci

We analyzed the co-localization between miRNAs and retroviral inserts mapped outside of CISs. These non-CIS inserts are non-clustered retroviral integration sites obtained in cancer screens. Their role (if any) in tumorigenesis is unclear and so these inserts are generally omitted from analysis. Low saturation in some cancer screens suggests that some non-CIS inserts might be located in regions involved in tumorigenesis, but on the other hand it is possible to speculate that some are just by-products of the cancer screen.

The number of miRNA located at a given distance from non-CIS inserts increases proportionally to the distance between the miRNA and non-CIS insert (R^2^ = 0.9396), whereas the number of miRNAs associated with CIS inserts does not display such a strong linear dependence (Fig. 2) (R^2^ = 0.7831). The association of miRNA with CIS inserts is better described by a logarithmic trendline with R^2^ = 0.945 (see Materials and Methods). The bootstrap simulation showed a significant enrichment for miRNA loci associated with non-CIS inserts, especially for distances less than 5 kb (Table 3). The number of non-CIS retroviral integration sites in the vicinity of miRNAs has a slightly higher enrichment than the enrichment for miRNA loci. Close examination of miRNA loci associated with non-CIS inserts revealed several loci with two independent non-CIS inserts located very close to each other. For example, among 13 miRNA loci (16 miRNAs) with non-CIS inserts within 10 kb, ten loci have a single insert, and three loci have two inserts. These closely located inserts were isolated from different cancer models, which is why these inserts were classified as non-CIS. We analyzed the distribution of distances between non-CIS inserts in the genome. There is significant increase in the number of non-CIS inserts located within 3 kb of each other, whereas the number of non-CIS insertions at longer distances is more or less uniform when measured in 1 kb bins. A total of 332 out of 3119 inserts outside of CISs are located within 3 kb of each other.

**Table 3:** Bootstrap analysis for co-localization between miRNAs and all non-CIS retroviral integration sites.

Distance, Kb	MiRNA loci, #	Observed # loci # inserts	Enrichment, Fold	Bootstrap (10^7^ iterations)	
				P	Max
5	245	10	3.3	0.0011	15
		12	3.7	0.00044	19
10	237	13	2.3	0.0054	20
		16	2.6	0.0018	26
20	233	25	2.3	0.000076	30
		30	2.5	0.000058	38
30	228	31	2.0	0.00018	38
		40	2.4	0.00001	47

See Table 2 for description of the columns.

We removed all non-CIS inserts located within 3 kb of each other and repeated the bootstrap analysis. Nevertheless, even this dataset shows approximately two-fold enrichment for miRNA loci associated with non-CIS inserts separated by 3 kb or more (Table 4). The enrichment is similar for all distances analyzed but the association at longer distances is statistically more significant.

Based on this bootstrap analysis we conclude that miRNA loci show the strongest association with CIS inserts at short distances (less than 10 kb). At distances less than 10 kb the enrichment of miRNA loci overlapping with CIS inserts is two times higher than the enrichment of miRNA loci overlapping with non-CIS inserts. At longer distances, such as 30 kb, miRNA loci show a similar association both with CIS and non-CIS inserts.

**Table 4:** Bootstrap analysis for co-localization between non-CIS inserts separated by more than 3 kb and miRNAs.

Distance, kb	MiRNA loci, #	Observed # loci	Enrichment, Fold	Bootstrap (10^7^ iterations)	
				P	Max
5	245	7	2.4	0.027	16
10	237	10	1.9	0.046	20
20	233	22	2.2	0.00058	30
30	228	29	2.0	0.00029	37

See Table 2 for description of the columns.

### MicroRNA loci are enriched around starts and ends of protein-coding genes

It is known that integration of murine leukemia viruses and MLV-derived vectors preferentially occurs around promoter regions [23]. Indeed, out of 3119 non-CIS retroviral sites from RTCGD database, 1190 (38%) are located within 5 kb of annotated transcription start sites of RefSeq genes (occupying ∼6.8% of the genome). This represents more than 5-fold enrichment, higher than the enrichment of non-CIS insertions around miRNA loci (Tables 3 and 4). Out of 2373 CIS inserts, 989 (42%) are located within 5 kb from annotated transcription start sites of RefSeq genes.

We analyzed the distribution of miRNA loci in the mouse genome with respect to RefSeq genes. Out of 381 miRNA locations, 155 (41%) overlap RefSeq Genes which occupy 32% of the mouse genome. Among these, 22 (6%) miRNAs overlap exons (2% of the genome), and 133 (35%) are located in introns (30% of the genome). Somewhat surprisingly, we found that miRNAs are enriched close to the start or the end of genes: 69 (18%) and 72 (19%) miRNAs are located within 5 kb of RefSeq gene transcription start or end sites, respectively (∼2.6 and ∼2.8 fold enrichment). In total, 105 (51%) miRNA locations are located within 5 kb either from the start, end, or both (>3-fold enrichment). Moreover, miRNAs show slightly higher enrichment in regions where gene start sites are separated from gene end sites by less than 10 kb (data not shown).

MicroRNAs associated with non-CIS inserts tend to be close to promoters of RefSeq genes while CIS-associated miRNAs tend to be distant from promoters. For example, 13 miRNA loci (16 miRNAs) have non-CIS inserts mapped within 10 kb. Out of these, 9 loci (69%) are located within 10 kb from RefSeq gene starts, while out of 10 CIS-associated miRNA loci only 4 are less than 10 kb from RefSeq gene starts. Six CIS-associated miRNA loci containing 17 miRNAs are located more than 10 kb away from promoters of RefSeq genes, indicating that the observed association between miRNAs and CISs is not explained by co-localization of miRNAs near genes. A similar tendency is observed for miRNA 5k loci (miRNAs within 5 kb of each other): out of 10 miRNA 5k loci with non-CIS RIS within 5 kb (Table 3), 7 overlap RefSeq gene starts. Out of 7 miRNA 5k loci with CIS RIS within 5k (Table 2), 3 overlap RefSeq gene starts.

Interestingly, human miRNAs are also enriched around transcription start or end sites of RefSeq genes. There are 543 annotated miRNAs in the human genome mapped to 474 unique miRNA precursor locations. Out of these 474 miRNA locations 108 (23%) are located within 5 kb of RefSeq gene transcription start or/and end sites (2.2-fold enrichment). We merged these 474 locations into 311 miRNA 5k loci by adding 5 kb on each side of the precursor and then created the base-pair-wise union (OR) of locations. Out of 311 miRNA 5k loci 92 (30%) overlap either transcription start or end sites of RefSeq genes, or both. These 92 loci contain 110 (23%) miRNA precursors. It seems that miRNA loci in the vicinity of transcription start or end sites contain less miRNA precursors than miRNA loci located farther from genes (1.2 and 1.7 miRNA precursors per loci, respectively).

### CIS-associated miRNAs are conserved and their human orthologs are located in cancer-associated regions

CIS-associated miRNAs have some common features. Most (21 out of 22) CIS-associated miRNAs are located outside of RefSeq protein-coding genes. The exception, *mir-135a-1*, is located in the 3′ UTR of the gene 6230410P16Rik. In contrast, 5 out of 16 miRNAs having non-CIS inserts within 10 kb are located within RefSeq genes. On average, CIS-associated miRNA loci contain more miRNAs than non-CIS associated miRNA loci (2.2 and 1.2 miRNA per locus, respectively).

CIS-associated miRNAs tend to be more conserved than miRNAs associated with non-CIS inserts, both in alignments of multiple vertebrate species and in human and mouse pairwise comparisons. First, we used pre-calculated phastCons [24] conservation scores based on 17 vertebrates for whole miRNA precursor sequences. The average conservation score for 22 CIS-associated miRNA is 0.941 versus 0.739 for 16 non-CIS-associated miRNA (see Materials and Methods for details). Second, we compared the sequence identity between mouse miRNA precursors and their orthologous human sequences. CIS-associated mouse miRNA precursors have 96% identity with human sequences whereas non-CIS-associated miRNAs display 91% identity, slightly lower than the average identity level for all mouse miRNA precursors (92%). In addition, CIS-associated miRNA precursors have significantly less indels between mouse and human sequences.

Considering the high conservation of CIS-associated miRNAs we looked for the involvement of human homologs in cancer development. Human homologs of eight CIS-associated miRNA loci are located in fragile regions and regions involved in cancer [7] (Table 1). It has been shown that miRNAs are generally down-regulated in cancer [8]. We therefore compared the available data on tissue-specific expression of human miRNAs [25] and the type of cancer associated with the CISs (blood or brain cancer) located in the vicinity of homologous murine miRNAs. Eight CIS-associated miRNA loci are co-localized with insertions determined from various types of blood cancer (Table 1). Human expression data in 24 different human organs and cell types was available for orthologs of members of 7 these loci [25]. There is a high correspondence between the miRNA expression pattern and the type of cancer induced by retroviral insertion near these miRNAs. Five loci have human miRNA orthologs whose expression is highest in tissues associated with blood development such as bone marrow or thymus. Another miRNA, *miR-22*, is enriched in thymus although it is most abundant in skeletal muscles [25].

## Discussion

Here we demonstrate that murine miRNAs are associated with CISs. The enrichment of miRNAs loci in proximity to CIS inserts is higher than the enrichment of miRNAs around non-clustered retroviral insertions located outside of CISs. All but one CIS-associated miRNA are located outside RefSeq genes and some of them may be classified as proto-oncogenes or tumor-suppressor genes. Indeed, the well-characterized oncogenic miRNA cluster *mir-17-92* [9], [26] is associated with CIS inserts (Table 1). Ectopic expression of the mir-17–92 cluster accelerates tumor development in a mouse B-cell lymphoma model [9]. Another example is the *mir-106a* miRNA cistron which shows numerous retroviral integrations in a lymphocyte tumor screen: tumors containing inserts close to the *mir-106a* miRNA cluster exhibit up to a 20-fold higher expression of these miRNAs [27]. There is also some evidence of the involvement of *mir-142* in cancer development [28].

Formally, we cannot exclude the possibility that insertions affect (distant) regulatory elements situated in *cis* to protein-coding genes, especially when the CISs occur relatively close to genes, e.g., in *HOX* clusters (Fig. 1c). However, an equally if not more plausible explanation is that retroviral insertions are causing changes in the expression of miRNA genes, especially in those cases where the insertions occur in close proximity to miRNAs, rather than affecting more distant protein-coding genes. For example, all five CIS insertions in region XqA5 are less than 5 kb from the three miRNAs listed but more than 120 kb from the assigned RIS gene *Gpc3*, and four out of seven CIS insertions in region 11qC are closer to *mir-142* than to the nearest assigned gene *Supt4h2*. It is also possible that that (some) miRNA genes have chromatin structure open to retroviral integration similar to promoter regions of protein coding genes. The CIS-associated miRNAs represent prospective candidates for further experimental studies in the context of cancer association.

It appears that miRNAs are enriched around the transcription start and/or end sites of protein-coding genes both in human and mouse. Considering the strong bias of MLV integrations around promoter regions [23] it is possible to speculate that the observed enrichment of non-CIS retroviral insertions near miRNAs may be at least partially due to the preferential location of miRNAs around promoter regions. Interestingly, miRNAs located close to transcription start or end sites tend to be non-clustered indicating a different type of organization of these miRNA genes.

## Materials and Methods

We used miRBase release 9.0 (October 2006) which contains 373 mouse miRNAs, 363 of which were mapped to 381 locations within the Mouse Feb 2006 (mm8) genome assembly (Build 36 “essentially complete” assembly by NCBI).

The July 2006 version of the Retroviral Tagged Cancer Gene Database (RTCGD, http://rtcgd.ncifcrf.gov) [19] contains 2373 retroviral integration sites classified as belonging to Common Integration Sites (CISs), and 3119 retroviral integration sites classified as located outside of CISs.

All analysis was done on a local mirror of the UCSC Genome Browser [20].

Bootstrap analysis was done similarly to [29]. Briefly, miRNAs were merged into miRNA loci by adding 5, 10, 20 or 30 kb on each side with subsequent base-pair-wise union (OR) on the UCSC Genome Browser. The resulting loci were randomly placed on the mouse genome with mapped CIS inserts or inserts outside of CISs and any cases of overlap were counted. Genome assembly gaps were removed from the analysis. Overlaps between randomly placed loci were prohibited. For each bootstrap test we performed 10^7^ iterations.

The conservation of mouse miRNAs was estimated using phastCons conservation scores [24] for whole miRNA precursors. The phastCons conservation scores were based on mouse-centric alignments of 17 species and were obtained from the UCSC genome browser [20]. The average phastCons score for bases within each known miRNA was calculated using the UCSC hgWiggle utility and the -doStats flag. Mouse-human base-pair identity scores were calculated using pairwise genome alignments obtained from the UCSC genome browser and the UCSC utilities axtAndBed and axtCalcMatrix.

The enrichment of miRNAs around transcription start or end sites was calculated as follows: all RefSeq gene transcription start and end sites were extracted from the genome browser. Annotations were created by adding 5 or 10 kb to every transcription start or end site. The enrichment was calculated as the ratio between the fraction of miRNAs overlapping these annotations and the fraction of the genome occupied by the corresponding annotations.

The linear and logarithmic trendlines and R2 values were calculated using Microsoft Excel 2003. The linear trendline for miRNAs associated with non-CIS inserts was described by y = 1.2286x+6.3333 with R^2^ = 0.9396. The linear trendline for miRNAs associated with CIS inserts was described by y = 0.3371x+17.6 and R^2^ = 0.7831. The logarithmic trendline for miRNAs associated with CIS inserts was described by y = 5.2282Ln(x)+9.3527 with R^2^ = 0.945.
